# Supplementary material for: Knowledge, attitude and practices among Healthcare Workers towards Pulmonary Plague Infection following an outbreak in Madagascar, 2017: A pilot study
Source: PLoS One. 2025 Dec 5;20(12):e0336589. doi: 10.1371/journal.pone.0336589 (PMC12680261; doi:10.1371/journal.pone.0336589)
Supplement: Attachment — Submitted filename: PONE-D-25-02320_reviewer.pdf [file pone.0336589.s002.pdf]

PLOS ONE

Knowledge, Attitude and Practices among Healthcare Workers towards Pulmonary Plague Infection following an Outbreak in Madagascar, 2017

--Manuscript Draft--

**Manuscript Number:**	PONE-D-25-02320
**Article Type:**	Research Article
**Full Title:**	Knowledge, Attitude and Practices among Healthcare Workers towards Pulmonary Plague Infection following an Outbreak in Madagascar, 2017
**Short Title:**	KAP amongst HCW towards Pulmonary Plague Infection following an outbreak in Madagascar, 2017
**Corresponding Author:**	Thomas Paerisch, MD<br>Robert Koch Institute: Robert Koch Institut<br>Berlin, GERMANY
**Keywords:**	Pneumonic Plague; Healthcare Workers; Yersinia pestis; Madagascar
**Abstract:**	Objectives<br><br>To assess training needs of healthcare workers (HCWs) on pulmonary plague (PP) control after the large PP outbreak in Madagascar 2017.<br><br>Methods<br><br>In 2018, we conducted a knowledge, attitudes and practices (KAP) survey among HCWs (PP cases and comparison group) in Antananarivo and Toamasina. Proportions were calculated, differences between groups were tested for significance.<br><br>Results<br><br>Among 59 HCW over 90% named the distinctive disease forms of plague (bubonic (93%), pulmonary (98%)), and 71% the causative agent. Washing hands was mentioned as protective measure by 58%, while 93% reported to have always washed hands after performing medical procedures. Only 3.5% reported managed PP cases before the outbreak; 38% reported to have felt confident performing invasive procedures while caring for PP cases at the beginning versus 62% at the end of the outbreak. HCW who remained uninfected reported more often than PP cases to have worn multiple use medical coats ((93% versus 53%, p=0.001), and less frequently to have paid for chemoprophylaxis out of pocket (13% versus 50%; p=0.01).<br><br>Conclusion<br><br>Despite the good overall knowledge, the mismatch between knowledge and practice of basic hygiene measures and low confidence in providing care for PP cases after the outbreak indicate a persisting need for training.
**Order of Authors:**	Thomas Paerisch, MD
	Inessa Markus
	Lynn Meurs
	Rebekah Wood
	Vaoary Razafimbia
	Andrianasolo Radonirina
	Delphin Kolie
	Rapelerano Rabenja Fahafahantsoa
	Alexandre Delamou
	Jan Walter
	Matthias Borchert

Powered by Editorial Manager® and ProduXion Manager® from Aries Systems Corporation

Additional Information:	
Question	Response
**Financial Disclosure**<br><br>Enter a financial disclosure statement that describes the sources of funding for the work included in this submission. Review the submission guidelines for detailed requirements. View published research articles from *PLOS ONE* for specific examples.<br><br>This statement is required for submission and **will appear in the published article** if the submission is accepted. Please make sure it is accurate.<br><br>**Funded studies**<br>Enter a statement with the following details:<br>• Initials of the authors who received each award<br>• Grant numbers awarded to each author<br>• The full name of each funder<br>• URL of each funder website<br>• Did the sponsors or funders play any role in the study design, data collection and analysis, decision to publish, or preparation of the manuscript?<br><br>Did you receive funding for this work?	Yes
Please add funding details.<br>as follow-up to "**Financial Disclosure**<br><br>Enter a financial disclosure statement that describes the sources of funding for the work included in this submission. Review the submission guidelines for detailed requirements. View published research articles from *PLOS ONE* for specific examples.<br><br>This statement is required for submission and **will appear in the published article** if the submission is accepted. Please make sure it is accurate.	Research for this publication was funded through the Global Health Protection Programme (GHPP) by the Federal Ministry of Health, Germany

**Funded studies**<br>Enter a statement with the following details:<br>• Initials of the authors who received each award<br>• Grant numbers awarded to each author<br>• The full name of each funder<br>• URL of each funder website<br>• Did the sponsors or funders play any role in the study design, data collection and analysis, decision to publish, or preparation of the manuscript?<br><br>Did you receive funding for this work?"	
Please select the country of your main research funder (please select carefully as in some cases this is used in fee calculation).<br>as follow-up to "**Financial Disclosure**<br><br>Enter a financial disclosure statement that describes the sources of funding for the work included in this submission. Review the submission guidelines for detailed requirements. View published research articles from *PLOS ONE* for specific examples.<br><br>This statement is required for submission and **will appear in the published article** if the submission is accepted. Please make sure it is accurate.<br><br>**Funded studies**<br>Enter a statement with the following details:<br>• Initials of the authors who received each award<br>• Grant numbers awarded to each author<br>• The full name of each funder<br>• URL of each funder website<br>• Did the sponsors or funders play any role in the study design, data collection and analysis, decision to publish, or preparation of the manuscript?<br><br>Did you receive funding for this work?"	GERMANY - DE
**Competing Interests**	The authors have declared that no competing interests exist

Use the instructions below to enter a competing interest statement for this submission. On behalf of all authors, disclose any competing interests that could be perceived to bias this work—acknowledging all financial support and any other relevant financial or non-financial competing interests.<br><br>This statement is required for submission and **will appear in the published article** if the submission is accepted. Please make sure it is accurate and that any funding sources listed in your Funding Information later in the submission form are also declared in your Financial Disclosure statement.<br><br>View published research articles from *PLOS ONE* for specific examples.<br><br>**NO authors have competing interests**<br><br>Enter: *The authors have declared that no competing interests exist.*<br><br>**Authors with competing interests**<br><br>Enter competing interest details beginning with this statement:<br><br>*I have read the journal's policy and the authors of this manuscript have the following competing interests: [insert competing interests here]*<br><br>* typeset	
**Ethics Statement**<br><br>Enter an ethics statement for this submission. This statement is required if the study involved:<br><br>• Human participants<br>• Human specimens or tissue<br>• Vertebrate animals or cephalopods<br>• Vertebrate embryos or tissues	Human participants were included.<br>Written informed consent was obtained from study participants.<br>Ethics clearance was obtained from the Committee of Ethics in Biomedical Research of the Ministry of Health in Madagascar on 30 April 2018 (File No. 048-MSANP/CERBM). A permission to conduct the study within the public health care setting was obtained by the national Ministry of Health of Madagascar. Data protection clearance was granted at Robert Koch Institute, Germany.

Powered by Editorial Manager® and ProduXion Manager® from Aries Systems Corporation

• Field research<br><br>Write "N/A" if the submission does not require an ethics statement.<br><br>General guidance is provided below. Consult the submission guidelines for detailed instructions. **Make sure that all information entered here is included in the Methods section of the manuscript.**	

**Format for specific study types**<br><br>**Human Subject Research (involving human participants and/or tissue)**<br>• Give the name of the institutional review board or ethics committee that approved the study<br>• Include the approval number and/or a statement indicating approval of this research<br>• Indicate the form of consent obtained (written/oral) or the reason that consent was not obtained (e.g. the data were analyzed anonymously)<br><br>**Animal Research (involving vertebrate animals, embryos or tissues)**<br>• Provide the name of the Institutional Animal Care and Use Committee (IACUC) or other relevant ethics board that reviewed the study protocol, and indicate whether they approved this research or granted a formal waiver of ethical approval<br>• Include an approval number if one was obtained<br>• If the study involved *non-human primates*, add *additional details* about animal welfare and steps taken to ameliorate suffering<br>• If anesthesia, euthanasia, or any kind of animal sacrifice is part of the study, include briefly which substances and/or methods were applied<br><br>**Field Research**<br><br>Include the following details if this study involves the collection of plant, animal, or other materials from a natural setting:<br>• Field permit number<br>• Name of the institution or relevant body that granted permission	
**Data Availability**<br><br>Authors are required to make all data underlying the findings described fully available, without restriction, and from the time of publication. PLOS allows rare exceptions to address legal and ethical concerns. See the PLOS Data Policy and FAQ for detailed information.	Yes - all data are fully available without restriction

A Data Availability Statement describing where the data can be found is required at submission. Your answers to this question constitute the Data Availability Statement and **will be published in the article**, if accepted.<br><br>**Important:** Stating 'data available on request from the author' is not sufficient. If your data are only available upon request, select 'No' for the first question and explain your exceptional situation in the text box.<br><br>Do the authors confirm that all data underlying the findings described in their manuscript are fully available without restriction?	
**Describe where the data may be found in full sentences. If you are copying our sample text, replace any instances of XXX with the appropriate details.**<br><br>• If the data are **held or will be held in a public repository**, include URLs, accession numbers or DOIs. If this information will only be available after acceptance, indicate this by ticking the box below. For example: *All XXX files are available from the XXX database (accession number(s) XXX, XXX.).*<br>• If the data are all contained **within the manuscript and/or Supporting Information files**, enter the following: *All relevant data are within the manuscript and its Supporting Information files.*<br>• If neither of these applies but you are able to provide **details of access elsewhere**, with or without limitations, please do so. For example:<br><br>*Data cannot be shared publicly because of [XXX]. Data are available from the XXX Institutional Data Access / Ethics Committee (contact via XXX) for researchers who meet the criteria for access to confidential data.*<br><br>*The data underlying the results presented in the study are available from (include the name of the third party*	The data underlying the results presented in the study are available from the corresponding author.

and contact information or URL).<br>• This text is appropriate if the data are owned by a third party and authors do not have permission to share the data.<br><br>* typeset	
Additional data availability information:	

Powered by Editorial Manager® and ProduXion Manager® from Aries Systems Corporation

Manuscript Click here to access/download;Manuscript;Manuscript_V4.docx

Knowledge, Attitude and Practices among Healthcare Workers towards Pulmonary Plague Infection following an Outbreak in Madagascar, 2017

Inessa Markus[1,2¶], Lynn Meurs[1,2¶], Rebekah Wood[3], Vaoary Razafimbia[4], Andrianasolo Radonirina[5], Delphin Kolie[6], Rapelerano Rabenja Fahafahantsoa[5], Alexandre Delamou[7], Jan Walter[1], Matthias Borchert[3¶¶,] Thomas Paerisch[8¶¶],

[1] *Department of Infectious Disease Epidemiology, Robert Koch Institute, Berlin, Germany*

[2] *ECDC Fellowship Programme, Field Epidemiology path (EPIET), European Centre for Disease Prevention and Control (ECDC), Solna, Sweden*

[3] *Centre for International Health Protection, Robert Koch Institute, Berlin, Germany*

[4] *Direction de la Veille Sanitaire et de la Surveillance Epidémiologique, Ministère de la Santé Publique (DVSSE), Antananarivo, Madagascar*

[5] *Laboratoire d'Accueil et de Recherche en Santé Publique et en Technologies de l'Information Médicale et de la Communication (LARTIC), Antananarivo, Madagascar*

[6] *Centre National de Formation et de Recherche en Santé Rurale de Maferinyah, Forécariah, Guinea*

[7] *Department of Public Health, Gamal Abdel Nasser University of Conakry, Conakry, Guinea*

[8] *Centre for Biological Threats and Special Pathogens, Robert Koch Institute, Berlin, Germany*

[¶] *Equal first author contribution;* [¶¶] *Equal last author contribution*

Corresponding author:

Thomas Paerisch (paerischt@rki.de; +49 30 18754-3108)

Robert Koch Institute

Centre for Biological Threats and Special Pathogens

Nordufer 20

13353 Berlin

The corresponding author will handle correspondence concerning the article, press proofs, and orders for off-prints.

Abstract

Objectives:

To assess training needs of healthcare workers (HCWs) on pulmonary plague (PP) control after the large PP outbreak in Madagascar 2017.

Methods:

In 2018, we conducted a knowledge, attitudes and practices (KAP) survey among HCWs (PP cases and comparison group) in Antananarivo and Toamasina. Proportions were calculated, differences between groups were tested for significance.

Results:

Among 59 HCW over 90% named the distinctive disease forms of plague (bubonic (93%), pulmonary (98%)), and 71% the causative agent. Washing hands was mentioned as protective measure by 58%, while 93% reported to have always washed hands after performing medical procedures. Only 3.5% reported managed PP cases before the outbreak; 38% reported to have felt confident performing invasive procedures while caring for PP cases at the beginning versus 62% at the end of the outbreak. HCW who remained uninfected reported more often than PP cases to have worn multiple use medical coats ((93% versus 53%, p=0.001), and less frequently to have paid for chemoprophylaxis out of pocket (13% versus 50%; p=0.01).

Conclusion:

Despite the good overall knowledge, the mismatch between knowledge and practice of basic hygiene measures and low confidence in providing care for PP cases after the outbreak indicate a persisting need for training.

Keywords: Pneumonic Plague, Healthcare Workers, *Yersinia pestis*, Madagascar

Introduction

Plague is considered endemic only in a few regions of the world. Single cases or small outbreaks were reported in the United States of America and in Kyrgyzstan, but the most affected countries according to WHO are the Democratic Republic of Congo, Peru and Madagascar. Over 75% of plague cases worldwide occur in Madagascar [1]. Until 2017, plague cases in Madagascar occurred mostly in rural regions, with a seasonal increase from October to April. Between 1998 and 2016, 88% of the cases reported were classified as bubonic plague (BP) [2]. Despite effective antibiotic treatment being available, prevention and control of the disease remain challenging, leading to its endemic persistence [3-5]. The large outbreak in 2017 was unusual in that it occurred between July and November, produced a large proportion of pulmonary plague (PP) cases, and affected urban areas like Antananarivo and Toamasina. 1878 of the 2412 notified cases (78%) in this time period were classified as PP [6], including 81 HCWs who were diagnosed with and treated for PP according to WHO [7]. Due to potential close contact with infected patients, healthcare workers (HCWs) are at high risk during outbreaks of PP, but only limited literature exists on transmission patterns and risk factors for infection among HCWs, and on their knowledge of the disease and treatment procedures [8-10]. Given the relatively high number of PP cases among HCWs reported during the 2017 Malagasy outbreak, we aimed at investigating possible knowledge gaps, unhelpful attitudes and unsafe practices during the outbreak that may have facilitated transmission to HCWs in order to better understand possible causes for

infection and develop locally relevant infection prevention and control (IPC) training to be better prepared for future plague outbreaks.

KAP surveys are used to assess knowledge, attitude and practices (KAP) of a selected population regarding a specific topic. They are commonly used in the health sector to gather before-and-after data on public health interventions [11-13]. For emerging diseases in limited resource settings, KAP surveys can help to develop targeted interventions addressing the most relevant problems and improve response measures in case of an outbreak. For example, during the Ebola Virus Disease outbreak in West Africa, KAP surveys contributed to describing and understanding the persisting perceptions and beliefs around the disease in the community [14-16].

Only limited literature on experiences of HCW on treatment of pulmonary plague in hospital setting. Therefore, we conducted the study to gather more information to support the development of context-adapted strategies for diagnosis, treatment and prevention of nosocomial transmission, with sufficient resources, including trained HCWs available on different levels of care.

Materials and methods

Study design:

In September 2018, we conducted a cross-sectional survey in hospitals of two Malagasy cities, Antananarivo and Toamasina, which were the most affected urban areas during the outbreak. The KAP survey was conducted in HCWs from the hospital departments where PP cases had arisen among HCWs that were involved in PP treatment, and from mobile teams that were involved in community outreach and body management of patients who died due

to PP in the 2017 outbreak. Recruitment of study participants was realized between 13 and 27 August 2018.

For the larger study of 81 HCW PP cases reported by WHO [7] 29 were selected from the national Directorate of Health and Epidemiological Surveillance (DVSSE) database based on geographical location (Antananarivo and Toamasina) and work in a health care facility at the point of study in 2018. These were contacted and invited to participate in the study. Additional HCWs were recruited as a comparison group from the same departments of hospitals where the HCW PP cases worked at the time of the study. HCWs were defined as medical doctors, nursing staff (nurses, midwives, and paramedics), cleaning personnel, medical interns and students. Administrative staff, for whom no patient contact was assumed, were excluded. Comparison HCWs were systematically sampled from staff lists to recruit 33% of each department’s HCWs. If a HCW was selected but absent for a long period of time, for example for holidays, another HCW was randomly sampled from the list. Upon written informed consent, local interviewers administered a structured questionnaire in French or Malagasy in a designated room to ensure privacy. The participants completed the interview in the language of their choice. Study participants received a token of appreciation for their participation (10 000 Ariary phone credit, equivalent to approx. € 2.50).

Data collection:

The questionnaire consisted of five sections. The first three sections applied to all participants and included questions about (1) demographic data (age, sex, work place, profession, and years of working experience), (2) knowledge (causative agent, transmission, IPC, treatment) and (3) attitudes towards PP (level of confidence in treatment and protection measures and material). HCWs that had worked in the hospital or a mobile unit

during the epidemic in 2017 also filled in the 4th section, with questions on practices during the epidemic (existing guidelines, availability of chemoprophylaxis, protection material and treatment). High-risk procedures were defined as suction, intubation, sputum sampling and cleaning of surfaces. Examples for invasive procedures, such as drawing blood and delivery, and examples for non-invasive procedures, such as performing ultrasound or ECG, were provided in the questionnaire. A sheet with pictures of the personal protection equipment was used to assess which items were used. The 5th section was only filled in by HCWs that were notified as a PP case during the outbreak, and contained questions about the disease, such as symptoms, onset of symptoms and treatment. At the end of the interview all HCWs were asked to demonstrate how to wear a surgical mask, which was assessed by a member of the research team.

Data analysis and definitions:

Throughout the epidemic, different case definitions for plague were applied. The DVSSE database we used for recruiting study participants included 29 HCW PP based on clinical, epidemiological and laboratory criteria.

After double data entry and validation, we used STATA 15 (StataCorp LLC, College Station, Texas, USA) for data management and analysis. A descriptive analysis was performed using frequency counts (n) and proportions (%).

We looked at differences in self-reported practice during the outbreak between HCW PP cases from the DVSSE database irrespective of the classification as suspected, probable or confirmed case with the comparison group. Due to the changes in the case definition and its unclear effect on the classification of cases in the database, as well as anecdotal evidence of problems with transport of samples and on-site testing with rapid diagnostic tests, we

conducted the same comparison on the subset of PCR-confirmed cases versus the corresponding subset of HCWs who worked in the same departments as the PCR positive cases. None of the cases had a positive culture and only one case had a positive serology result recorded in the database.

For the comparison of proportions, Pearson chi^2-tests and Fisher's exact tests were performed as appropriate. We applied a significance level of $p<0.05$, and did not correct for multiple testing since we conducted an explorative study and did not test for any predefined hypothesis.

Results

Characteristics of the study population

From the 29 HCW PP cases from the national DVSSE case database 15 HCW could be traced back and agreed to participate. These HCW were working in seven different institutions (hospitals and mobile teams) in Antananarivo and Toamasina, where an additional 44 HCWs were recruited as a comparison group, resulting in a total of 59 participants. The remaining 14 HCW PP cases had changed positions or hospitals after the outbreak, were on leave during the study, or refused to participate and could therefore not be included in the study.

From the 59 HCW, 54% were working in Antananarivo and 46% in Toamasina at the time of the survey. More female (63%) than male HCW (37%) were in the sample with an age range of 22 to 59 years. Most participants were nurses (41%), or doctors including medical interns (41%). Eleven HCWs (19%) were support personnel (cleaning, patient transport or morgue staff, guard or mobile community outreach team). The majority of HCWs (78%) reported to have more than five years of working experience, and most confirmed to have received a

salary (92%); four volunteers reported to not have received any financial compensation during the outbreak (Table 1).

Table 1: Demographic characteristics of study participants (n=59, unless stated otherwise)

	All participants (n=59)	PP cases HCW (n=15)	HCW comparison group (n=44)
**City**			
Antananarivo	32 (54%)	5 (33%)	27 (61%)
Toamasina	27 (46%)	10 (67%)	17 (39%)
**Gender**			
male	22 (37%)	7 (47%)	15 (34%)
female	37 (63%)	8 (53%)	29 (66%)
Age range (years)	22 - 59	22 - 58	24 - 59
**Profession**			
MD	24 (41%)	7 (47%)	17(39%)
Paramedic	24 (41%)	6 (40%)	18(41%)
Other staff	11 (19%)	2 (13%)	9 (21%)
**Working experience (years)**			
< 2 years	6 (10%)	2 (13%)	4 (9.1%)
2-5 years	10 (17%)	2 (13%)	8 (18%)
>5 years	43 (78%)	11 (73%)	32 (73%)
**Received salary during the outbreak**	54 (92%)	12 (80%)	42 (96%)

From the 15 HWCs recorded as PP cases, 6 HCWs had a positive PCR confirming the infection according to the DVSSE database. Five HCWs were from Antananarivo and 10 HCW from Toamasina. The distribution of professional groups among HCW PP cases and the HCW of the comparison group was comparable. The age among the 15 HCW PP cases ranged from 22 to 58 years and among the comparison group from 24 to 59 years.

Knowledge on pulmonary plague

Almost all participants were aware that PP was a contagious disease (98%) to which no immunity is developed (83%, Table 2).

178 **Table 2: General knowledge among HCWs concerning the disease, prevention and treatment (n=59, unless stated**
179 **otherwise)**

**To which extent do you agree with the following statement:** **"Pneumonic plague is a contagious disease"**	
Some agreement	1 (1.7%)
Total agreement	58 (98%)
**To which extent do you agree with the following statement:** **"Certain people are immune towards PP"**	
Does not know	3 (5.1%)
Does not agree	49 (83%)
Some agreement	2 (3.2%)
Total agreement	6 (10%)
**What is the pathogen of PP? (n=58)**	
Does not know	4 (6.9%)
Virus	11 (19%)
Parasite	1 (1.7%)
Bacteria	42 (71%)
**What are different clinical forms of plague? (Please mention the types you know)**	
Bubonic	55 (93%)
Pulmonary	58 (98%)
Primary or secondary pulmonary	4 (6.8%)
Septicemic	12 (20%)
**What are the (main) symptoms of PP? (Please mention the one you know)**	
Fever	56 (95%)
Dyspnea	23 (39%)
Coughing	47 (80%)
Chest pain	20 (34%)
Expectorations	46 (78%)
**Speaking of prevention methods against PP in hospitals, can you tell me which ones you know?**	
Isolation	44 (76%)
Washing hands	33 (58%)

Chemoprophylaxis	39 (67%)
Wearing a surgical mask (patients)	40 (68%)
Wearing a mask (HCW)	55 (93%)
Wearing gloves	47 (80%)
**Can you name a drug to take in case of chemoprophylaxis?**	
Co-trimoxazole	49 (83%)
Doxycycline	39 (66%)
Ciprofloxacin	18 (31%)

Most participants knew that the plague is caused by bacteria (71%), but some also thought that it is caused by a virus (19%). Few HCWs mentioned the septicemic form of plague (20%) and certain symptoms of PP (chest pain 34%, dyspnea 39%). Most frequently mentioned protective measures were HCWs wearing a mask (93%) and using gloves (80%). Most HCWs knew the following preventive measures: isolating patients (76%), asking patients to wear a mask (68%), taking chemoprophylaxis (67%), and washing hands (58%). When asked about chemoprophylaxis, a majority of HCWs specified co-trimoxazole (83%), followed by doxycycline (66%) and ciprofloxacin (31%).

Attitudes

The reported confidence in terms of treating patients increased over the course of the outbreak: the proportion of HCWs who reported to have felt totally confident treating PP cases including invasive procedures rose from 38% at the beginning of the outbreak to 62% at its end (Table 3).

Table 3: Attitudes towards PP among HCW (n=59, unless stated otherwise)

	Total agreement	Some agreement	No agreement		I don't know
**Indicate the extent to which you agree with the following statements:**					
"I was confident in performing the high-risk procedures at the beginning of the 2017 epidemic" (n=50)	19 (38%)	7 (14%)	23 (46%)		1 (2.0%)
"I was confident in performing the high-risk procedures towards the end of the 2017 epidemic" (n=50)	31 (62%)	9 (18%)	10 (20%)		

	Expert knowledge	Intermediate knowledge	Basic knowledge	No knowledge	I don't know
How would you describe your knowledge of prevention of nosocomial/hospital transmission of PP? (n=58)	6 (10%)	32 (55%)	15 (26%)	3 (5.2%)	2 (3.5%)

	Very easy/ Easy	Average	Difficult	Very difficult	I don't know
How easy is it to protect yourself from transmission of pneumonic plague in a hospital setting?	27 (46%)	11 (19%)	14 (24%)	7(12%)	
How easy is it to treat patients with pneumonic plague in a hospital setting?	4 (6.8%) / 29 (49%)	6 (10%)	17 (29%)	1 (1.7%)	2 (3.4%)

	Total agreement	Some agreement	No agreement		I don't know
**Indicate the extent to which you agree with the following statement:**					
"I trust the personal protective equipment." (n=58)	33 (57%)	17 (29%)	8 (14%)		

"I trust the drugs for chemoprophylaxis."	39 (66%)	18 (31%)	2 (3.4%)	
"I was afraid of contracting pneumonic plague outside of work during an epidemic."	39 (66%)	3 (5.1%)	17 (29%)	
"I was afraid of getting infected with PP at work (in the hospital) during an outbreak."	40 (68%)	4 (6.8%)	14 (24%)	1 (1.7%)
"During the epidemic the workload was too high" (n=57)	45 (79%)	4 (7.08%)	8 (14%)	
"During the epidemic we were understaffed." (n=57)	36 (63%)	3 (5.3%)	18 (32%)	
	**Yes**	**No**	**I don't know**	
Are health workers (in general) sufficiently informed about the transmission of PP?	42 (71%)	14 (24%)	3 (5.1%)	

A small majority of participants (55%) described their level of knowledge of how to prevent nosocomial transmission of PP as intermediate, and only 10% felt to have expert knowledge at the time of the study. A majority felt that HCW in general were sufficiently informed about the transmission routes of PP (71%). Additionally, 46% considered it to be easy to protect themselves in a hospital setting, and 55% considered it to be easy or very easy to treat PP patients. Only 57% of participants trusted protective equipment fully, while 66% fully trusted chemoprophylaxis. About two thirds reported to have feared contracting PP at work (68%) or outside work (66%). A majority of respondents reported a high workload (79%), and felt that the departments were understaffed during the outbreak (63%).

Practices

The most common clinical practices were local disinfection (39%), clinical examinations (42%) and invasive procedures (39%). Among high-risk procedures the most common were surface cleaning in treatment centers (37%), followed by sputum sampling (32%) and intubation (25%). Most HCWs (91%) reported to have followed guidelines during the outbreak (Table 4).

Table 4: Self-reported practices of HCW during the plague epidemic in 2017 (n=59, unless stated otherwise)

**Use of PPE**	**Always**	**Most of the time**	**Sometimes**	**Never**	**I don't know**
Surgical mask worn by patients (n=57)	31 (54%)		16 (28%)	10 (18%)	
Surgical mask worn by HCW while caring for patients (n=57)	26 (46%)		17 (30%)	13 (23%)	1 (1.8%)
FFP2 mask worn by HCW while caring for patients (n=57)	26 (46%)		26(46%)	5 (8.8%)	
Gloves (n=57)	49 (86%)		8 (14%)		
Coat (multiple use) (n=57)	47 (80%)		3 (5.1%)	7 (12%)	
Coat (single use) (n=57)	28 (49%)		17 (30%)	12 (21%)	
**Protective practices**					
Did you wash your hands after performing high-risk procedures? (n=56)	52 (93%)	4 (7.1%)			
Were the patients with PP isolated? (n=57)	55 (97%)	1 (1.8%)		1 (1.8%)	
**Use of guidelines/ protective measures**	**Yes**	**No**			**I don't know**

Adherence to guidelines/protocols for management PP cases and prevention of infection with PP (n=57)	54 (91%)	2 (3.4%)		1 (1.7%)
During the epidemic did you take any medication (chemoprophylaxis) to prevent infection with PP? (n=54)	49 (91%)	5 (9.3%)		
Co-trimoxazole (n=42)	32 (76%)	10 (24%)		
Doxycycline (n=43)	25 (58%)	18 (42%)		
Ciprofloxacin	7 (12%)	52 (88%)		
Did you pay out of pocket for chemoprophylaxis? (n=52)	12 (23%)	40 (77%)		
Did you pay out of pocket for protective equipment (PPE) (n=52)	12 (23%)	40 (77%)		
Did you take care of/treated people with PP that you knew personally? (n=55)	27 (49%)	28 (51%)		
**Clinical practice**				
Clinical examination	25 (42%)	34 (58%)		
Non-invasive care	8 (14%)	51 (86%)		
Invasive care	23 (39%)	36 (61%)		
Local disinfection	23 (39%)	36 (61%)		
Transport of sick patients	10 (17%)	49 (83%)		
Dead body management	9 (15%)	50 (85%)		
Suction	1 (1.7%)	58 (98%)		
Intubation*	15 (25%)	44 (75%)		
Sputum sampling*	19 (32%)	40 (68%)		

Cleaning of surfaces in a treatment center*	22 (37%)	37 (62%)	
**Participation in PP training and experience in care for PP cases**			
Did you ever manage cases of PP before the 2017 outbreak? (n=58)	2 (3.5%)	55 (95%)	1 (1.7%)
Did you participate in PP courses/trainings before the outbreak? (n=58)	12 (21%)	46 (79%)	
Did you participate in PP courses/trainings during the outbreak? (n=58)	29 (50%)	27 (47%)	2 (3.5%)
Did you participate in PP courses/trainings after the outbreak? (n=58)	12 (21%)	44 (76%)	2 (3.5%)
Did you participate in PP courses/trainings at any point in time? **	42 (71%)	17 (29%)	
	**Correct**	**Wrong**	
Demonstration of use of a surgical masque (n=59)	30 (52%)	28 (48%)	

* high-risk procedures

** This variable was calculated based on the answers given to participation in training before/during/after the outbreak.

A small majority (54%) of respondents stated that the patients always wore surgical masks during medical care. In regards to their own practice, 46% of the participants responded that they always wore a surgical mask, and 46% to have always worn an FFP2 mask while caring for patients. A large majority of the participants reported to have always worn gloves (86%) and to have always used a multiple use coat (80%).

Most HCWs (93%) reported to have always washed their hands after high-risk procedures (Table 5).

Table 5: Differences between PP cases and comparison group among HCW in self-reported practices during the plague epidemic in 2017 (n=59, unless stated otherwise)

	**Cases**	**Non-cases**	**p-value**
**Did you follow guidelines/protocols for the management of patients with PP and prevention of infection with PP during the epidemic in 2017? (n=57)**			0.066
Yes	13 (87%)	41 (98%)	
No	2 (13%)	0	
I don't know	0	1 (2.4%)	
**Which of the following personal protective equipment (PPE) did you use with pneumonic plague cases during the 2017 outbreak:**			
**Surgical mask worn by patients (n=57)**			0.992
Always	9 (60%)	22 (52%)	
Sometimes	4 (27%)	12 (29%)	
Never	2 (13%)	8 (19%)	
**Surgical mask worn by HCW while caring for patients (n=57)**			0.582
Always	9 (60%)	17 (41%)	
Sometimes	4 (27%)	13 (31%)	
Never	2 (13%)	11 (26%)	
I don't know	0 (%)	1 (2.4%)	
**FFP2 mask worn by HCW while caring for patients (n=57)**			0.739
Always	7 (47%)	19 (45%)	
Sometimes	6 (40%)	20 (48%)	
Never	2 (13%)	3 (7.1%)	
**Gloves (n=57)**			0.188

Always	11 (73%)	38 (91%)	
Sometimes	4 (27%)	4 (9.5%)	
**Coat (multiple use) (n=57)**			0.001
Always	8 (53%)	39 (93%)	
Sometimes	3 (20%)	0	
Never	4 (27%)	3 (7.1%)	
**Coat (single use) (n=57)**			0.028
Always	3 (20%)	25 (60%)	
Sometimes	7 (47%)	10 (24%)	
Never	5 (33%)	7 (17%)	
**Overalls *(combinaison)* (n=57)**			0.448
Always	3 (20%)	4 (9.5%)	
Sometimes	4 (27%)	10 (24%)	
Never	8 (53%)	28 (67%)	
**Do you wash your hands after performing high-risk procedures? (n=56)**			1
Always	14 (93%)	38 (93%)	
Most of the time	1 (6.7%)	3 (7.3%)	
**Were the patients with PP isolated? (n=57)**			0.461
Always	14 (93%)	41 (97%)	
Most of the time	0 (%)	1 (2.4%)	
Never	1 (6.7%)	0	
**During the epidemic, did you take any medication (chemoprophylaxis) to avoid infection with PP? (n=54)**			1
Yes	12 (92%)	37 (90%)	
No	1 (7.7%)	4 (9.8%)	
**Co-trimoxazole (n=42)**			0.404
Yes	9 (90%)	23 (72%)	
No	1 (10%)	9 (28%)	
**Doxycycline (n=43)**			1
Yes	7 (58%)	18 (58%)	

No	5 (42%)	13 (42%)	
**Ciprofloxacin (n=59)**			1
Yes	2 (13%)	5 (11%)	
No	13 (87%)	39 (89%)	
**Did you pay out of pocket for chemoprophylaxis? (n=52)**			0.010
Yes	7 (50%)	5 (13%)	
No	7 (50%)	33 (87%)	
**Did you pay out of pocket for protective equipment (PPE) (n=56)**			1
Yes	3 (21%)	9 (24%)	
No	11 (79%)	29 (76%)	
**Did you take care of people with PP that you knew personally? (n=55)**			0.768
Yes	8 (53%)	19 (48%)	
No	7 (47%)	21 (53%)	
**Did you ever manage cases of PP before the 2017 outbreak? (n=58)**			1
Yes	0 (0%)	2 (4.7%)	
No	15 (100%)	40 (93%)	
I don't' know	0	1 (2.3%)	
**Did you participate in PP courses/trainings before the outbreak? (n=58)**			0.156
Yes	1 (6.7%)	11 (26%)	
No	14 (93%)	32 (74%)	
**Did you participate in PP courses/trainings during the outbreak? (n=58)**			1
Yes	8 (53%)	21 (49%)	
No	7 (47%)	20 (47%)	
I don‘t know	0 (0%)	2 (4.7%)	
**Did you participate in PP courses/trainings after the outbreak? (n=58)**			0.312

Yes	5 (33%)	7 (16%)	
No	10 (67%)	34 (79%)	
I don't know	0 (0%)	2 (4.7%)	
**Did you participate in PP courses/trainings at any point in time? * (n=59)**			1
Yes	11 (73%)	31 (71%)	
No	4 (27%)	13 (30%)	
**Demonstration of use of a surgical masque (n=58)**			0.373
Correct	9 (60%)	19 (44%)	
Wrong	6 (40%)	24 (56%)	

Patients with PP were almost always isolated according to participants (97%). The use of chemoprophylaxis during the epidemic was common among the participants (91%). The most common antibiotic used was co-trimoxazole (76%). A minority of participants paid out of pocket for the chemoprophylaxis (23%), or for protective material (23%). 49% of the HCWs reported to have taken care of a PP case that they knew personally.

Only 2 HCWs (3.5%) had previous experience in working with PP-cases before the 2017 epidemic. A minority of the HCWs reported to have had PP classes or training before the epidemic in 2017 (21%). During the epidemic 50% received training. After the epidemic, 21% attended training on health care provision for PP patients (Table 4). In total, 29% of HCW had not received any PP training, and a small majority of participants (52%) could correctly demonstrate the use of a surgical mask.

Differences between PP cases and comparison group

When the 15 HCW PP cases were compared to the 44 participants of the comparison group, we found that most differences between the two groups were not statistically significant. A

significantly lower proportion of the cases compared to the comparison group reported to always have worn the multiple use medical coats (53% versus 93%, p=0.001) or a single use medical coat (20% versus 60%; p= 0.028). A significantly higher proportion of the cases reported to have paid for chemoprophylaxis out of their own pockets (50% versus 13%; p=0.01). Other practices did not significantly differ between PP cases and the comparison group (Table 5).

The analysis of the subset of the six PCR confirmed cases and their comparison group of 26 HCWs also indicated a similar significant difference for reported usage of multiple use medical coats (50% versus 100%; p=0.004), and a significantly higher proportion of PCR confirmed cases received training after the outbreak (67% versus 20%; p=0.043).

Discussion

This study provides information on knowledge, attitudes and practices during the plague outbreak in 2017 and highlights the gaps in IPC training and implementation of IPC measures among HCW in urban areas in Madagascar. We found basic PP knowledge to be good. Prevention measures like wearing masks and gloves and isolating patients were well known. Some theoretical knowledge, however, was lacking, such as: knowing about the pulmonary and bubonic but not the septicemic form of plague, knowing the unspecific symptom "fever" but not common symptoms "dyspnea" or "chest pain". HCWs felt confident about their abilities to treat patients and perceived a learning effect by working during the outbreak. Nonetheless, the results indicate room for improvement, and there remain training needs concerning disease characteristics, necessary IPC measures and their purpose, and the correct use of personal protective equipment.

There were several discrepancies between knowledge and reported practice. First of all, transmission prevention measures like hand washing after providing care for a PP case were mentioned by only half of the HCWs, but when asked about hand hygiene directly, virtually all reported to have practiced it during the epidemic after performing a high-risk procedure. This gap between knowledge and reported practice suggests that HCWs' reported practices may be overstated compared to actions performed in reality. Alternatively, they may have practiced hand hygiene more often than naming it as preventive measure, suggesting that it may not have been fully understood why certain procedures are important while caring for PP cases. Conversely, a higher proportion of HCWs knew that suspected cases should wear a mask (68%), but a lower proportion reported that this was done in practice (54%). Also, wearing a mask by HCWs themselves was mentioned by 93% as a protective measure, while only half of them reported to have always worn a surgical or FFP2 mask (46% and 46%, respectively) while caring for patients. In addition, only 52% could demonstrate how to correctly wear a surgical mask. This gap between knowledge and practice in relation to patients may indicate a lack of implementation of, or compliance with, guidelines despite better knowledge, or insufficient availability of protective equipment such as masks. The gap may also be due to the fact that only few HCWs had training before or after the epidemic on handling PP patients. Further training, availability of biosafety guidelines and access to sufficient PPE are likely to increase staff safety and confidence [17-19].

PP cases more often reported out-of-pocket payment of chemoprophylaxis than HCWs from the comparison group. This may suggest that cases have experienced financial barriers to chemoprophylaxis and were, therefore, less well protected against infection. We therefore recommend ensuring equal access to chemoprophylaxis for all HCWs.

The high-risk of contracting PP from working in the hospital or mobile team in the community was perceived as similar to contracting PP from outside the work context. The analysis of 2414 plague cases, 78% PP cases and 16% BP cases, between August and November 2017 did not reveal any recorded nosocomial transmission [6]. Our study population seemed to have good knowledge of various aspects of the disease, including its clinical manifestations, and of adequate infection prevention practices. While only a small proportion of the HCWs had received training during and after the epidemic, and a considerable proportion had not received any training at all, the hands-on experience may have led to reported practice being better than theoretical knowledge, and to confidence to treat patients with PP to grow in the course of the epidemic. Worryingly, only half of the HCW (52%) could demonstrate the correct use of a surgical mask, which suggests a persisting need for hands-on training.

The case definition changed over the evolution of the outbreak, which may have contributed to misclassification if HCW were not trained accordingly. Lack of knowledge of case definitions, possibly in combination with lack of knowledge and training on PP, may have contributed to possible misclassification of HCW as PP cases. A sensitive case definition ensured the capture of all suspected cases of PP, which is desirable due to the fast evolution and severity of disease. However, with limited capacity for adequate testing despite having samples from the majority of cases, only 2% of the clinically suspected PP cases in 2017 could be laboratory confirmed [6]. Consequently, the number of PP cases may have been overestimated.

This study has various limitations. We interviewed the HCWs in their working environment, and although we ensured privacy for the interview of each HCW they may have been reluctant to voice criticism or report mismanagement for fear of retributions. Courtesy and

recall bias may have embellished the answers on practices during the outbreak one year before this study, while knowledge and attitude were assessed one year after the outbreak. Changes of case definition and availability and reliability of diagnostic tests over the course of the epidemic may have influenced the inclusion of PP cases in the DVSSE database. The re-identification of cases one year after the epidemic for the larger study was performed by another research team. Due to the selection process we could only include a part of the HCW PP cases that were selected from the DVSSE database within a larger study, remained in the same unit after the outbreak and were available at the time of the study. This may have led to a biased selection of PP cases.

Conclusions

This study shows that one year after the 2017 plague epidemic, HCWs in urban areas in Madagascar had a good level of knowledge, but only half reported to feel confident treating PP cases despite the hands-on experience during the outbreak. Our results indicate that there may be a persistent need among HCWs for training in IPC methods, such as hand hygiene, PPE use, and adequate isolation, to protect both HCW and patients. Despite awareness of existing guidelines, standard operating procedures for PP-specific PPE-use and management of PP cases tailored to the Malagasy setting may contribute to being better prepared for future large-scale outbreaks.

Acknowledgements

We would like to thank the health care workers who dedicated, despite their heavy workload, significant time to the study team and to make this survey possible. Furthermore, we would like to extend our gratitude to the DVSSE, LARTIC and Pasteur Institute for their invaluable contribution to this study.

References

1. World Health Organization. Plague around the world, 2010–2015. Releve epidemiologique hebdomadaire. 2016;91(8):89-93. doi:10.1016/j.actatropica.2012.04.013.

2. Andrianaivoarimanana V, Piola P, Wagner DM, Rakotomanana F, Maheriniaina V, Andrianalimanana S, et al. Trends of Human Plague, Madagascar, 1998-2016. Emerging infectious diseases. 2019;25(2):220-8. doi:10.3201/eid2502.171974.

3. Rabaan AA, Al-Ahmed SH, Alsuliman SA, Aldrazi FA, Alfouzan WA, Haque S. The rise of pneumonic plague in Madagascar: current plague outbreak breaks usual seasonal mould. Journal of medical microbiology. 2019;68(3):292-302. doi:10.1099/jmm.0.000915.

4. Riehm JM, Löscher T. [Human plague and pneumonic plague : pathogenicity, epidemiology, clinical presentations and therapy]. Bundesgesundheitsblatt, Gesundheitsforschung, Gesundheitsschutz. 2015;58(7):721-9. doi:10.1007/s00103-015-2167-9.

5. Stenseth NC, Atshabar B, Begon M, Belmain S, Bertherat E, Carniel E, et al. Plague past present future. PLoS Med. 2008;5(1):e3. doi:10.1371/journal.pmed.0050003.

6. Randremanana R, Andrianaivoarimanana V, Nikolay B, Ramasindrazana B, Paireau J, Ten Bosch QA, et al. Epidemiological characteristics of an urban plague epidemic in

Madagascar, August-November, 2017: an outbreak report. The Lancet Infectious diseases. 2019;19(5):537-45. doi:10.1016/s1473-3099(18)30730-8.

7. World Health Organization. Plague Outbreak Madagascar. WHO external Situation report 14. 14 ed: World Health Organization; 2017. p. 9. Available from: http://apps.who.int/iris/bitstream/handle/10665/259556/Ex-PlagueMadagascar04122017.pdf?sequence=1 Access Date: 16.05.2024.

8. Kool JL. Risk of person-to-person transmission of pneumonic plague. Clinical infectious diseases : an official publication of the Infectious Diseases Society of America. 2005;40(8):1166-72. doi:10.1086/428617.

9. Mitchell FK. The plague in Cape Town in 1901 and its subsequent establishment as an endemic disease in South Africa. SA Medical Journal 1983;64:17-9.

10. Evans C. Pneumonic Plague: Incidence, Transmissibility and Future Risks. Hygiene. 2022;2(1):14-27.

11. Katler Q, Godiwala P, Macri C, Pineles B, Simon G, Chang A, et al. Evolution of Knowledge, Awareness, and Practices regarding Zika Virus from 2016 to 2017. Infectious Diseases in Obstetrics and Gynecology. 2017;2017:6350602. doi:10.1155/2017/6350602.

12. Otu A, Ebenso B, Okuzu O, Osifo-Dawodu E. Using a mHealth tutorial application to change knowledge and attitude of frontline health workers to Ebola virus disease in Nigeria: a before-and-after study. Human Resources for Health. 2016;14(1):5. doi:10.1186/s12960-016-0100-4.

13. Lin Y, Huang L, Nie S, Liu Z, Yu H, Yan W, et al. Knowledge, Attitudes and Practices (KAP) related to the Pandemic (H1N1) 2009 among Chinese General Population: a Telephone Survey. BMC Infectious Diseases. 2011;11(1):128. doi:10.1186/1471-2334-11-128.

14. Iliyasu G, Ogoina D, Otu AA, Dayyab FM, Ebenso B, Otokpa D, et al. A Multi-Site Knowledge Attitude and Practice Survey of Ebola Virus Disease in Nigeria. PloS one. 2015;10(8):e0135955. doi:10.1371/journal.pone.0135955.

15. Jalloh MF, Bunnell R, Robinson S, Jalloh MB, Barry AM, Corker J, et al. Assessments of Ebola knowledge, attitudes and practices in Forécariah, Guinea and Kambia, Sierra Leone, July-August 2015. Philosophical transactions of the Royal Society of London Series B, Biological sciences. 2017;372(1721):20160304. doi:10.1098/rstb.2016.0304.

16. Ngatu NR, Kayembe NJ, Phillips EK, Okech-Ojony J, Patou-Musumari M, Gaspard-Kibukusa M, et al. Epidemiology of ebolavirus disease (EVD) and occupational EVD in health care workers in Sub-Saharan Africa: Need for strengthened public health preparedness. Journal of epidemiology. 2017;27(10):455-61. doi:10.1016/j.je.2016.09.010.

17. Allegranzi B, Kilpatrick C, Storr J, Kelley E, Park BJ, Donaldson L. Global infection prevention and control priorities 2018-22: a call for action. Lancet Global Health. 2017;5(12):e1178-e80. doi:10.1016/s2214-109x(17)30427-8.

18. World Health Organization. Guidelines on core components of infection prevention and control programmes at the national and acute health care facility level. World Health Organization; 2016. p. 90. Available from: https://apps.who.int/iris/handle/10665/251730 Access Date: 16.05.2024.

19. World Health Organization. Interim practical manual: supporting national implementation of the WHO guidelines on core components of infection prevention and control programmes. . World Health Organization; 2017. p. 77. Available from: https://apps.who.int/iris/handle/10665/330073 Access Date: 16.05.2024.
