# Supplementary material for: Knowledge, attitude and practices among Healthcare Workers towards Pulmonary Plague Infection following an outbreak in Madagascar, 2017: A pilot study
Source: PLoS One. 2025 Dec 5;20(12):e0336589. doi: 10.1371/journal.pone.0336589 (PMC12680261; doi:10.1371/journal.pone.0336589)
Supplement: Attachment — Submitted filename: Response to reviewers.docx [file pone.0336589.s004.docx]

Dr Thomas Paerisch

[Thomas-Kratz@posteo.de](mailto:Thomas-Kratz@posteo.de)

To the Editor, PLOS ONE.

8 October 2025

RE: RESPONSE TO REVIEWER’S COMMENTS: MANUSCRIPT NO.: PONE-D-25-02320

Dear Sir or Madam,

Many thanks to you for your helpful feedback. We have tried to address all your concerns to the best of our abilities. Kindly help us with your further concerns (if any) to improvise our paper. Below we are providing our responses to the comments and actions that we took to address them. The responses are indented and highlighted in blue.

========

Reviewer #1: Knowledge, Attitude and Practices among Healthcare Workers towards Pulmonary

Plague Infection following an Outbreak in Madagascar, 2017

The study aimed to assess training needs of healthcare workers (HCWs) on pulmonary plague (PP) control after the Madagascar 2017 large PP outbreak. The authors in 2018, conducted a knowledge, attitudes and practices (KAP) survey among HCWs (PP cases and comparison group) in Antananarivo and Toamasina.

General comments: The study is described as comparative between PP cases and the comparison group, but the rationale for the sample size selection needs further clarification. The study includes only 15 PP cases and 44 comparison group participants, which may limit the representativeness of the results for the entire population of PP cases. Therefore, this study should be presented as a pilot study, due to the small sample size. I therefore suggest the following title: "Knowledge, Attitude and Practices among Healthcare Workers towards Pulmonary Plague Infection following an Outbreak in Madagascar, 2017 : A pilot study ".

Response: Accepted, title changed as suggested: "Knowledge, Attitude and Practices among Healthcare Workers towards Pulmonary Plague Infection following an Outbreak in Madagascar, 2017: A pilot study"

The demographic data are presented for PP cases and the comparison group, but not all results are compared between PP cases and the comparison group. The methodology requires further clarification to ensure the rigor and reproducibility of the study.

Response: We accept the criticism have modified the tables and now systematically compare HCW PP cases and HCW comparison group in Tables 2 to 4.

The percentages shown in the tables are often inconsistent, sometimes totaling less than or exceeding 100%.

Response: Indeed there have been a few miscalculations, so we have reviewed the percentages and believe that in general, they now add up to 100%, where appropriate.

However, we have opted for the following rounding and reporting policy for better readability: we have opted for limiting the number of reported digits to two meaningful digits, e.g. 45%, 4.5%, 0.45%. For better transparency we have added the following explanation to the methods section: "For better readability, we limit the reporting of digits to two meaningful digits, as in 45%, 4.5% or 0.45%. As a result of this rounding policy, percentages may add up to slightly less or more than 100%."

The authors note that the study shows that one year after the 2017 plague epidemic, HCWs in urban areas in Madagascar had a good level of knowledge. However, they do not specify the criteria used to determine a "good level of knowledge."

Response: We now specify the threshold for "good knowledge" to be 75% and above, and for poor knowledge to be below 40%. The relevant sentences in the results section read: "Prevention measures like wearing masks and gloves and isolating patients were well known (≥75% of participants). Some theoretical knowledge, however, was lacking, such as: knowing about the pulmonary and bubonic but not the septicemic form of plague, knowing the unspecific symptom "fever" but not common symptoms "dyspnea" or "chest pain" (<40% of participants)."

The authors should specify whether the data from this study are available, in a Data Availability section.

Response: Data availability section added, complying with journal requirements. It reads: “*Data availability*: The collected raw data is only accessible to the study team including the corresponding author. The study protocol, including its chapter on the informed consent and basis for the realized Ethical Review Board clearance, stipulates that collected raw data is treated confidentially. Furthermore, sharing raw data would entail the risk of indirect identifying due to the low number of study participants.

To improve the quality of the manuscript, we encourage the authors to incorporate the following suggestions.

ABSTRACT:

The abstract accurately reflects the objectives, findings, and provides a clear overview of the background, and key results. The results could be strengthened by emphasizing the comparison of plague knowledge between PP cases and the comparison group.

Response: Thanks for the comment. The abstract's result section now starts with the following statement: "Knowledge levels were similar for HCW PP cases and the HCW comparison group."

INTRODUCTION

The paper provides adequate background on plague, KAP (Knowledge, Attitudes, and Practices) surveys, and the 2017 outbreak in Madagascar.

Line 51: "Despite the good overall knowledge" What are the criteria used to define "good overall knowledge"? This should be clarified in the methodology section.

Response: In the methods section, we now write: "The threshold for a group having "good knowledge" was defined as ≥75%, while the threshold for a group "lacking knowledge" was defined as <40%."

Line 72: "Relatively high number of PP cases among HCWs reported during the 2017 Malagasy outbreak." Please specify the number or prevalence.

Response: The sentence now reads: " Given the relatively high number of PP cases among HCWs reported during the 2017 Malagasy outbreak (N=81), …"

Line 86: "Only limited literature on experiences of HCWs on treatment of pulmonary plague in hospital." Please cite some examples if they exist.

Response: Thank you for the suggestion. The following references have been inserted:
8.	Kool JL. Risk of person-to-person transmission of pneumonic plague. Clinical infectious diseases : an official publication of the Infectious Diseases Society of America. 2005;40(8):1166–72. doi:10.1086/428617.
17.	Salam AP, Raberahona M, Andriantsalama P, Read L, Andrianarintsiferantsoa F, Razafinambinintsoa T, et al. Factors Influencing Atypical Clinical Presentations during the 2017 Madagascar Pneumonic Plague Outbreak: A Prospective Cohort Study. The American journal of tropical medicine and hygiene. 2020;102(6):1309–15. doi:10.4269/ajtmh.19-0576.

MATERIALS AND METHODS

The methods are appropriate for the study’s objectives. The data collection and analysis techniques are generally well-described. However, I suggest adding a detailed explanation of the sample size calculation to enhance reproducibility.

Response: We have clarified the sample size for the group of HCW PP cases and for the comparison group as follows:

Concerning the number of participating HCW PP cases (N=15), we now write:
"From 81 HCW plague cases reported by WHO [7], 29 PP cases were selected from the Ministry of Health's national Directorate of Health and Epidemiological Surveillance (DVSSE) database, based on geographical location (Antananarivo and Toamasina) and work in a health care facility at the time of study in 2018. These were contacted and invited to participate in the study (Figure 1). … From the 29 eligible HCW PP cases from the national DVSSE case database 15 HCW could be traced back and agreed to participate (Figure 1)."

Concerning the comparison group, we now write:
"To avoid selection bias, comparison HCWs were systematically sampled from HCW staff lists of each eligible department. The sample size of N=44 was determined on feasibility grounds, resulting in a sampling fraction of 33% (Figure 1)."

Study design:

Line 93: "We conducted a cross-sectional survey in hospitals..." How many hospitals were included in the study?

Response: The requested information has been added; the sentence now reads: "… we conducted a cross-sectional survey in four hospitals in the Malagasy capital Antananarivo, and in two hospitals and one mobile team in the Malagasy city Toamasina …"

Line 98: "Recruitment of study participants was conducted between 13 and 27 August 2018." There was a one-year gap between the end of the 2017 epidemic and the surveys. This is therefore a retrospective study. Please clarify how recall bias was controlled during data collection.

Response: On minimizing courtesy and recall bias we now write: "We interviewed the HCWs in their working environment, and although we ensured privacy for the interview of each HCW to minimize courtesy bias, they may have been reluctant to voice criticism or report mismanagement for fear of retributions. Our study was carried out one year after the outbreak, and we used photographic prompts to minimize recall bias."

However, we frankly admit and discuss the possibility of bias as a limitation of our study: "Still, our study cannot distinguish with confidence between knowledge and attitude acquired during or after the outbreak, and residual courtesy and recall bias may have embellished the answers on practices during the outbreak."

Line 108: "Comparison HCWs were systematically sampled from staff lists to recruit 33% of each department’s HCWs." Why was this 33% proportion chosen? Is it supported by literature review? If so, please provide a reference.

Response: The sampling fraction of 33% was determined by resource availability. We clarify as follows concerning the HCW comparison group: "The sample size of N=44 was determined on feasibility grounds, resulting in a sampling fraction of 33% (Figure 1)."

Data collection:

Line 116: "The first three sections applied to all participants..." Further, in Line 120, "HCWs who had worked in the hospital or a mobile unit during the 2017 epidemic also completed the 4th section, which included questions on practices." Please specify how many participants completed each section.

Response: We apologize for the confusion we have created – Sections 1 to 4 have been completed by all participants.

We now write: "The same questionnaire was used on all participants. It consisted of four sections: (1) demographic data (age, sex, work place, profession, and years of working experience), (2) knowledge (causative agent, transmission, infection prevention and control, symptoms, treatment), (3) attitudes towards PP (level of confidence in treatment and protection measures and material). The 4^th^ section consisted of questions on practices during the epidemic (use of existing guidelines, availability of chemoprophylaxis, protection material and treatment). High-risk procedures were defined as suction, intubation, sputum sampling and cleaning of surfaces. Examples for invasive procedures, such as drawing blood and delivery, and examples for non-invasive procedures, such as performing ultrasound or ECG, were provided in the questionnaire. A sheet with photographs of the personal protection equipment was used to assess which items were used."

Lines 127-129: "The 5th section was only completed by HCWs who were notified as a PP case during the outbreak and contained questions about the disease, such as symptoms, onset of symptoms, and treatment." However, questions about symptoms and treatment are addressed in Table 2 for all 59 participants. Does this imply that additional analyses were conducted but are not presented in this article?

Response: Again, we apologize for the misunderstanding we have created. All 59 participants were asked about symptoms and treatments they KNOW – these results are reported in Table 2. In the 5^th^ section, the HCW PP cases only were asked which symptoms and treatments they have EXPERIENCED themselves – but this section is contribute to the KAP study reported in this paper. So we have now deleted the reference to the 5^th^ section and write: "The same questionnaire was used on all participants. It consisted of four sections … The 4^th^ section consisted of questions on practices during the epidemic (use of existing guidelines, availability of chemoprophylaxis, protection material and treatment). High-risk procedures were defined as suction, intubation, sputum sampling and cleaning of surfaces. Examples for invasive procedures, such as drawing blood and delivery, and examples for non-invasive procedures, such as performing ultrasound or ECG, were provided in the questionnaire. A sheet with photographs of the personal protection equipment was used to assess which items were used. At the end of the interview all HCWs were invited to demonstrate how to wear a surgical mask, which was assessed by a member of the research team."

RESULTS

The results are clearly presented but require a reorganization in how they are presented. The description of the tables should be provided in full, and the corresponding table should be inserted after the text. Currently, the authors partially describe the table, insert it, and then continue with the description. This approach disrupts the flow and should be revised to enhance clarity and readability.

Response: We agree with this comment, and have moved the tables to the end of the text.

Characteristics of the study population

Line 155: "These HCWs were working in seven different institutions..." Please specify these institutions. In Table 1, for the profession "MD" Please write out the full term (e.g., "Medical Doctor").

Response: The sentence now reads: "These HCW were working in seven different institutions in Antananarivo (four hospitals) and Toamasina (two hospitals, one mobile team)".

In Table 1, the acronym "MD" has been replaced by "Medical Doctors".

Lines 172-173: "The distribution of professional groups among HCW PP cases and the HCW of the comparison group was comparable." By "comparable," do you mean « similar »?

Response: Yes – the sentence now reads: "The distribution of professional groups among HCW PP cases and the HCW of the comparison group was similar."

Line 178, Table 2: The study is presented as a comparative analysis between PP cases and the comparison group. So, the authors should provide a comparative table between PP cases and the comparison group. If not, please specify in the methodology section, which analyses involve comparisons between PP cases and the comparison group and justify why comparisons are not made for all analyses.

Response: All tables now include comparisons between HCW PP cases and HCW comparison group.

Verify/Recalculate the proportions in Table 2. For example, "Some agreement" = 1 (1.7%) and "Total agreement" = 58 (98%), which totals 99.7% instead of 100%.

Response: To improve readability, we have limited the reporting of digits to two meaningful digits, as in 17%, 1.7%, 0.17% etc. This is why percentages may add up not exactly to 100%, but to slightly less or more than 100%, as in the example above.

For the question, "To what extent do you agree with the following statement: 'Certain people are immune towards PP'," the total is 101.3% instead of 100%.

Response: The percentages have been revised and now add up to 99.8%. They do not add up to 100% because we do not report decimals when the percentage is 10% or above – for better readability, as now explained in the methods section: "For better readability, we limit the reporting of digits to two meaningful digits, as in 45%, 4.5% or 0.45%. As a result of this rounding policy, percentages may add up to slightly less or more than 100%."

Tables 3 and 4: The authors should provide a comparative table between PP cases and the comparison group. Also, verify/recalculate the proportions in these tables.

Response: Tables 2, 3 and 4 have been revised and now provide comparisons between HCW PP cases and HCW comparison group. Percentages have been verified and corrected where necessary. However, as explained above, totals may still be slightly smaller than 100% because we round percentages to two meaningful digits.

Table 5: Verify/recalculate the proportions in the table. Many proportions are either above or below 100%.

Response: Table 5 has now been omitted, since its results are incorporated in Tables 2 to 4.

Lines 237-238: "After the epidemic, 21% attended training on health care provision for PP patients (Table 4)." Elements of interpretation for Table 4 should be included before Table 4, not after Table 5.

Response: All tables have been moved to the end of the document, as suggested. Table 5 has been omitted.

DISCUSSION

Line 257: "We found basic PP knowledge to be good" Specify in the methodology the criteria used to define good or poor knowledge in this study.

Response: In the methods section, we now write: "The threshold for a group having "good knowledge" was defined as ≥75%, while the threshold for a group "lacking knowledge" was defined as <40%."

=====

Reviewer #2: This manuscript addresses an important and underexplored topic regarding the knowledge, attitudes, and practices of healthcare workers (HCWs) during a pulmonary plague outbreak in Madagascar. The study design is valuable and the inclusion of diverse HCW roles—from doctors to cleaning staff—adds depth. I appreciate that the authors addressed comments from a previous version.

However, there are still sections that would benefit from clearer articulation, particularly around the description of the study groups and case definitions. The paragraph explaining the comparison of PCR-confirmed cases versus other HCWs, for instance, is essential but currently difficult to follow.

A diagram illustrating the sampling process and case categorization would greatly improve clarity. Additionally, some aspects of the questionnaire distribution across diverse HCW roles (e.g., clinical vs. non-clinical staff) could be justified further, especially if the same questions were applied across the board.

Response:

We have reworded the paragraph on PCR-confirmed cases as follows: "We conducted a subgroup analysis of six PCR confirmed cases and their corresponding comparison group of 26 HCWs. We found a significant difference for reported usage of multiple use medical coats (50% versus 100%; p=0.004). A significantly higher proportion of PCR confirmed cases than comparison HCW received training after the outbreak (67% versus 20%; p=0.043)."

We have added a diagram (Figure 1) explaining the recruitment process of HCW PP cases and HCW comparison group.

We clarify that "Eligible comparison HCWs were defined as medical doctors, nursing staff (nurses, midwives, and paramedics), cleaning personnel, medical interns and students. Administrative staff, for whom no patient contact was assumed, were excluded."

For the questionnaire use, we have added the following clarification: "The same questionnaire was used on all participants. It consisted of five sections."

I also suggest expanding the interpretation of findings regarding access to chemoprophylaxis. For instance, HCW PP cases may have sought medication on their own due to fear or perceived urgency, not only due to systemic barriers. Highlighting such nuances would strengthen the discussion.

Response: Thanks for this comment. We have expanded the paragraph accordingly, which now reads: "A fifth of the HCW participants report to have paid for chemoprophylaxis themselves. This is worrying, since chemoprophylaxis should be provided to exposed HCWs for free. Whether chemoprophylaxis was unavailable to these HCWs, or whether their initiative reflected their fears, could not be distinguished by our study. However, the difference between HCW PP cases and HCW comparison group was particularly striking in this respect: an almost five times higher proportion of cases reported out-of-pocket payment for chemoprophylaxis than HCWs from the comparison group. This may suggest that cases have experienced access barriers to chemoprophylaxis and were, therefore, less well protected against infection. We therefore recommend ensuring equal access to chemoprophylaxis for all exposed HCWs."
